# Vocalic Intrusions in Consonant Clusters in Child-Directed vs. Adult-Directed Speech

**DOI:** 10.3389/fpsyg.2021.688002

**Published:** 2021-07-19

**Authors:** Nina Gram Garmann, Pernille Hansen, Hanne Gram Simonsen, Elisabeth Holm, Eirik Tengesdal, Brechtje Post, Elinor Payne

**Affiliations:** ^1^Department of Early Childhood Education, OsloMet – Oslo Metropolitan University, Oslo, Norway; ^2^MultiLing – Center for Multilingualism in Society Across the Lifespan, Department of Linguistics and Scandinavian Studies, University of Oslo, Oslo, Norway; ^3^Department of Humanities, Inland Norway University of Applied Sciences, Hamar, Norway; ^4^Department of Linguistics and Scandinavian Studies, University of Oslo, Oslo, Norway; ^5^Faculty of Modern and Medieval Languages and Linguistics, University of Cambridge, Cambridge, United Kingdom; ^6^Faculty of Linguistics, Philology and Phonetics, University of Oxford, Oxford, United Kingdom

**Keywords:** child-directed speech (CDS), consonant clusters, language acquisition, Norwegian, prosodic-phonetic biases, vocalic intrusions

## Abstract

In this paper, we investigate a prosodic-phonetic feature in child-directed speech within a dynamic, complex, interactive theoretical framework. We focus on vocalic intrusions, commonly occurring in Norwegian word initial consonant clusters. We analysed child-directed speech from nine Norwegian-speaking mothers to their children, aged 2;6, 4, and 6 years, and compared the incidence and duration of vocalic intrusions in initial consonant clusters in these data with those in adult-directed speech and child speech. When viewed overall, vocalic intrusion was found to be similar in incidence in child- and adult-directed speech. However, closer examination revealed differential behaviour in child-directed speech for certain conditions. Firstly, a difference emerged for one particular phonetic context: While vocalic intrusions in /Cr/ clusters are *frequent* in adult-directed speech, their presence is *near-categorical* in child-directed speech. Secondly, we found that the duration of vocalic intrusions was longer in child- than in adult-directed speech, but only when directed to 2;6-year-olds. We argue that vocalic intrusions in child-directed speech may have both a bonding as well as a didactic function, and that these may vary according to the age of the child being addressed.

## Introduction

In infant- and child-directed speech (IDS and CDS), adults are known to adjust their speech in various ways. For example, IDS and CDS have shorter and less complex sentences (Snow, 1972) with fewer false starts and hesitations. Adults repeat their utterances more to 2-year-olds than to 10-year-olds (Snow, 1972), place key words at the end of an utterance, or sometimes in isolation, and produce them with more emphatic stress (Aslin et al., 1996). On a phonological level, IDS and CDS are reported to be generally more exaggerated in their intonation with a higher pitch and wider pitch range, and are slower in tempo (Cruttenden, 1994).

In a recent review article, Wang et al. (2018) discuss acoustic properties of IDS, that is, speech to children younger than 24 months. They report that a large body of literature shows that prosodic modifications such as higher pitch, larger pitch variability, slower tempo and longer vowel duration are attested in IDS when compared to ADS across a wide range of languages (Cristià, 2013). Concerning segmental properties, they report fewer studies and the findings are more mixed, possibly due to differences between languages. For instance, Kuhl et al. (1997) have reported a more expanded vowel space in IDS than in ADS (for American English, Russian and Swedish), indicating hyperarticulation, while others have found a reduced vowel space (Benders, 2013, for Dutch and Englund and Behne, 2006, for Norwegian), suggestive of hypoarticulation. For the Norwegian vowels /æ(:), ø(:), o(:), y(:), ʉ(:), e(:)/, Englund (2018) also found evidence for hypoarticulation in IDS compared to ADS, for example with more front articulation and less lip protrusion in IDS; the lack of rounding was possibly attributable to mothers smiling to their infants when talking to them. For consonants, VOT values have been found to increase (Englund, 2005, for Norwegian), to be maintained (Baran et al., 1977, for English) or to decrease (Sundberg and Lacerda, 1999, for Swedish) in IDS compared to ADS. Some studies have found consonants to be more clearly articulated in IDS than in ADS (Cristià, 2010; Dilley et al., 2014, for English), while the opposite has also been found (Martin et al., 2015, for Japanese).

Wang et al. (2018) lay out how IDS changes over time as the child develops and note that parents adjust both to the child’s chronological age, and also, in the case of cochlear implantation in children with hearing loss, to their peers matched in hearing experience. They conclude therefore that parents may modify their speech to children to adapt to the latter’s needs. When children are still young infants, prosodic exaggeration may be more important, while later on, it may be that other linguistic information, like segmental information, is of higher value to the child. Some characteristics of IDS disappear already during the child’s first year, while other characteristics may persist over a longer time span. Rattanasone et al. (2013) showed that IDS-specific tonal characteristics of Cantonese-speaking mothers’ speech to their infants had already disappeared at 12 months, whereas for example Stern et al. (1983) showed that the tonal characteristics of IDS in American English diminished over time, but that there were still differences between CDS and ADS at 24 months.

The findings of Wang et al. (2018) relate to IDS, that is, to speech addressed to infants in the earliest stages of their development. Although the literature is sparser on CDS than IDS, there is evidence that adults speak differently to children even when they are older than 24 months. In a study comparing mothers’ speech to children aged 2, 4, and 6 in Catalan, English and Spanish to speech addressed to adults, Payne et al. (2010) found that speech was both proportionally more vocalic and containing more even-timed vocalic intervals in CDS than in ADS, characteristics that were shown to mirror those of the children’s own speech. They also reported that these characteristics in the CDS did not change across the child age-span covered. Poulain and Brauer (2018) examined different aspects of child-directed communication to German children aged between 2 and 6 years: mean length of utterance, pointing, and variability of pitch. They found that the mothers adapted their behaviour to the advancing abilities of their children. As to prosody, variability of pitch decreased with age: there was a significant difference between ADS and CDS at 2, 3, and 5 years, but not at 6 years, so this phonetic adaptation disappeared sometime between 5 and 6 years. Comparing mothers’ and fathers’ speech to 2- and 5-year-olds with ADS, Warren-Leubecker and Bohannon (1984) found that mothers adopted a higher pitch when speaking to both 2-year-olds and 5-year-olds, but that they had a wider pitch range when speaking to the younger children. The fathers raised their pitch and increased their pitch range to the 2-year-olds, but did not adjust their pitch when speaking to 5-year-olds compared to when speaking to adults. This shows that mothers, at least, speak differently to their children even when the latter are older than 24 months, and also that there are changes in CDS as the child grows older.

In addition to investigating whether and how adults modify their speech when addressing infants and children, and whether and how this varies as a function of child age, research in this area has sought to identify the function(s) of CDS. Building on a large body of research, Wang et al.(2018, p. 19) conclude that IDS may have three possible functions: “to maintain infants’ attention, to communicate affect, and to be didactic.” According to Wang et al. (2018), there is evidence in favour of the hypothesis on adults using IDS because children are attracted to ‘happy’ speech that communicates affect. They claim that there is less evidence for a didactic function to the IDS register. However, there may be a difference in the function of IDS and CDS registers, as the characteristics of the dyadic relationship and communicative priorities shift. Fernald and Mazzie (1991) suggest that while IDS may have the function of drawing the child’s attention, the function of CDS may be more of a didactic one where more distinct speech may help the child to segment individual words from the stream of fluent speech. Even though children start the process of segmenting individual words from the speech stream earlier than from 24 months, this process may also be relevant later, and the distinct speech may serve to enhance morpho-syntactic and phonological characteristics of the words. Identifying possible functions is not a straightforward task, however, and one might be able to attribute various possible functions to a single characteristic. For example, the repetition of words and utterances in CDS may have a didactic function (e.g., to facilitate the learning of lexical items or word shape) as well as being a way of keeping the conversation running and linking it to the child’s interest, thus combining attention-seeking, the communication of affect and a didactic function.

Independently of the underlying function of these modifications, the evidence clearly shows that adults change the way they speak when addressing infants and young children as compared to when speaking to adults. As we have seen, these speech accommodations occur on different levels, that is, segmental phonetic, prosodic, or higher-level linguistic, and potentially with different degrees of speaker awareness. Certain properties of CDS may be characterised as relatively ‘local,’ for example the placement of greater emphasis on a given word, while others may be more pervasive throughout a stretch of speech, such as slower speech tempo. Some that may *appear* to be relatively local, for example a segmental difference in vowel quality, may actually arise from a more general effect of hyperarticulation, which results in an expanded vowel space and, in turn, in changed vowel qualities for individual vowels. Certain characteristics of CDS may be relatively language-independent, particularly those that are less didactic in purpose and more attributable to general, (quasi-)universal strategies of attracting and maintaining attention which we might plausibly assume to have adapted toward general properties of the (developing) human perceptual system. Other strategies may more deliberately draw attention to structural features that are specific to a given language, for example the exaggeration of geminate duration, thereby emphasising a lexical contrast, and we might plausibly attribute a more didactic motivation to these.

Any variation in the input, as characterised by modifications apparent in CDS, may potentially shape the child speech acquisition process, by shifting the distribution of patterns to which the child is exposed. Nevertheless, this is not taken to be a passive process. Vihman and Velleman argue that while patterns in the ambient language shape the acquisition process, the process is also influenced by the child’s phonetic skills and her own emerging phonological patterns: ‘the onset of phonological systematisation is superimposed upon ongoing phonetic learning’ (2000: 265, see also Vihman, 2017). This process of interaction between salient properties in the speech the child hears on the one hand, and the child’s own phonetic abilities and emergent phonological system on the other, may result in apparent discontinuities in development, with phonological structure arising from phonetic patterns in a way that is not only gradual. While a child’s first words may result from her matching her own productions to what she hears, (the ‘articulatory filter,’ Vihman, 1993), this is not purely a mechanical process; instead, ‘certain phonetic structures are exploited and generalised,’ in the formation of nascent phonological systems, or ‘templates,’ which may lead to non-adult-like adaptations. Thus, while guided by the child’s own phonetic development and the phonetic patterns in the input speech she is exposed to, the nascent phonological system exerts, in turn, its own pull on the child’s speech productions. As a result, any influence of particular CDS characteristics will itself be mediated through this self-organising process (cf. Davis and Bedore, 2013). The child does not simply mirror what the adult does. There is, instead, a dynamic interaction between the adult’s speech – with its own structures and patterns that may be modified in addressing the child – and the child’s emerging structures and speech patterns. For Davis and Bedore (2013), the acquisition process also involves a dynamic interaction between the input (speech input from communication partners as well as their extrinsic critical guidance, cf. the didactic purpose discussed above) and the child’s developing intrinsic biological and cognitive abilities, but their model places a greater emphasis on functional pressures arising from the child’s need to connect and communicate, and hence, they identify the child’s growing capacity for social interaction as a third key factor in child phonological development.

As well as being a dynamic process, which we imagine may shift according to discourse and wider context and as the child develops, this interaction is also complex, in that the structures in both the adult’s speech and the child’s speech are, inevitably, implemented phonetically, adding further scope for divergence. Languages, and language varieties, vary not only in their phonological structure, but also in how that structure is implemented. Thus, aside from features that are more clearly either phonological (e.g., pertaining to a lexical contrast or phonotactics) or general phonetic (e.g., pertaining to general articulatory skills), language-specific *linguistic-phonetic* features are also a body of knowledge to be acquired. These are phonetic (i.e., non-contrastive) aspects of a given language, or variety of language, that a child must master in order to be a native or near-native speaker, for example cross-linguistic variation in whether [s] is produced as laminal or apical, or in VOT for the cueing of voicing contrasts. Such linguistic-phonetic features may also pertain to temporal coordination of gestures and their association with prosody, and thus to the production of connected speech, in what can be characterised as prosodic-phonetic tendencies (or *biases*; see Payne, 2016, for a fuller discussion). Thus, prosodic-phonetic biases are pervasive, systematic and language-specific phonetic patterns in the implementation of phonological structure. Evident in adult-directed speech, they may be more or less salient to the listener, including the infant listener. As such, we can consider them as the language-specific phonetic ‘packaging’ which conveys phonological structure, and thus acquisition of that structure for a particular language is mediated via these patterns.

One example of a prosodic-phonetic bias in Norwegian are vocalic intrusions in the production of consonant clusters, the characteristics of which in ADS we briefly describe here. In contrast to most variants of English, Norwegian has a so-called open transition between consonants in a cluster, where the first consonant is released before the onset of the next one (Endresen, 1991, p.127, see also Bradley, 2007). This often leads to vocalic intrusions between a sequence of consonants in a cluster in adult speech (e.g., [1bᵊɭoː] for [1bɭoː] *blå*, ‘blue’). In a recent study, we found vocalic intrusions in 30.6% of instances for (Urban East) Norwegian, while for (Southern British) English, vocalic intrusions were nearly non-existent (affecting only 0.6% of instances) (Garmann et al., 2021). In the same study, vocalic intrusions were found to be more common in ADS when C2 is a liquid, with the greatest incidence occurring when C2 is a rhotic tap or flap, and a particularly high incidence when this was combined with a voiced stop in C1 position. We observed that ‘the incidence of vocalic intrusion in Norwegian is gradient and clearly shaped at least in part by articulatory considerations.’ (2021, p. 22). Hence, while not obligatory in any phonetic context, vocalic intrusions are nevertheless common in Norwegian, and neither their incidence nor duration are dependent on speech rate in ADS.

It is important to reflect here on what this means for diverging inputs into the child speech acquisition process. In terms of phonological structures, the evidence available in the input may be very similar (and indeed the phonotactics of English and Norwegian are quite similar). Thus, if the English-ambient child and the Norwegian-ambient child are exposed to similar input – at least with respect to this particular variable – their nascent phonological systems, and thus their own productions should also be fairly similar (at least no more dissimilar than between two children of the same ambient language). However, if we consider not just the phonological structures but also *how they are implemented*, we can model actual divergence in inputs, and make different predictions about the children’s early productions. Indeed, in the same study (Garmann et al., 2021), we found that, while vocalic intrusions were also evident in English child speech [as one possible strategy for tackling clusters, and one that is attested to some degree cross-linguistically (McLeod et al., 2001)], they were far less prevalent than in Norwegian child speech. This could indicate that in selecting a strategy for tackling clusters infants are influenced by distributional patterns in their ambient speech input. In other words, young children are sensitive to speech patterns of different degrees of granularity, and these include not just which segments can be juxtaposed in connected speech, and where, but crucially also *how* they are juxtaposed, that is, the fine detail of intersegmental coordination. Furthermore, these provide another type of evidence from which their own nascent systems are forged. Indeed, these vocalic intrusions were found to be even more frequent and longer in duration in Norwegian child speech than in Norwegian ADS, with some generalisation to phonetic contexts for which there was no incidence in ADS, for example /sC/ clusters. Together these pieces of evidence strongly suggest that vocalic intrusions, arising from a particular setting of temporal and articulatory coordination, are a pervasive *bias* (a strong but non-obligatory tendency) in the production of Norwegian consonant clusters, and one that shapes the acquisition pathway of Norwegian-ambient infants.

The fact that children generalise vocalic intrusion to other phonetic contexts suggests that *linguistic-phonetic knowledge at the implementation level* is part and parcel of the child’s nascent phonological system. Note that we are not suggesting that the child is interpreting the vocalic intrusion as having the status of a phonological segment – something which is theoretically possible but for which there is insufficient evidence. Rather, we are proposing that knowledge about the *implementation* of phonological structure should be seen as part of *knowledge about phonological structure*. And thus, a child’s nascent phonological system will also include knowledge of how that system is implemented, and just as the system itself may diverge from the adult system, so may (language-specific) properties relating to its implementation. In part this also depends on the distributional properties of the input, and indeed raises the question as to whether these biases may be subject to modification in CDS. If they are, we may also ask what form this modification might take, and to what extent such modification may be interpreted as *deliberate*, or *incidental*, as the unplanned consequence of slower speech tempo, for example. Here, we investigate the role that CDS may have in mediating the prosodic-phonetic bias of vocalic intrusion in Norwegian in the child acquisition process. In our earlier study, we found that child speech displays more vocalic intrusion in consonant clusters than does ADS (Garmann et al., 2021), which suggests vocalic intrusion may be more prevalent in CDS than in ADS. This could come about quite incidentally: since vocalic intrusions may be influenced by the slower tempo and exaggerated prosody that characterise CDS, we hypothesise that CDS has longer and more frequent vocalic intrusions than ADS. Even though we did not find any influence of speech rate on the incidence and duration of vocalic intrusions in ADS, slower speech rate is a known characteristic of CDS (Cruttenden, 1994) and could thus be a determining factor in the incidence of vocalic intrusions in CDS. In this scenario, a greater incidence of vocalic intrusions would simply emerge from other CDS behaviours. If based on these general properties of CDS, there would be no reason to expect any differences in the phonetic contexts in which the vocalic intrusions occur; on the contrary, we would expect the pattern of vocalic intrusions to be very similar to that in ADS, only with longer durations and potentially higher incidence. These would be the result of generalised CDS strategies which can be interpreted as having broader functions of increasing the infant’s attention, increasing closeness in the dyadic relationship (‘bonding’) and a general facilitation of comprehension (i.e., not focussing on any structures in particular). This would constitute a kind of speech accommodation that acknowledges the child’s different capacities and knowledge, and renders adult speech patterns more transparent (through slowing down and exaggeration).

Another scenario is that adults adjust the prevalence and/or distribution of vocalic intrusions when addressing children. Such changes would be difficult to attribute solely to broader, more generalised CDS strategies such as tempo, and would point to a more focussed and localised strategy (however, conscious or not) of speech accommodation. The potential reasons for doing so are multiple, and would likely affect to some degree the nature of the adjustments being made. One possibility is that adults accommodate toward the child’s own speech patterns. CDS would under this scenario show patterns of vocalic intrusion that more closely mirror those evident in child speech, for example through a greater incidence of intrusions and/or incidence in phonetic contexts in which intrusions are detected in child speech but not in ADS. Similarly with the previous scenario, this would also constitute a kind of speech accommodation that acknowledges the child’s different capacities and knowledge, but rather than rendering adult speech patterns more transparent, chooses to close the communicative gap by adjusting speech patterns *toward* those of the child. We would attribute this kind of accommodation to a desire to increase the closeness of the dyadic relationship (‘bonding’) and potentially to facilitate comprehension. A longer duration of intrusions could also fall under this scenario, since as well as an attention-calling device, it can also be seen as a mirroring of the child’s own productions.

Another possibility is that adults hyperarticulate consonant clusters to clarify the elements of the cluster in what is potentially an instructive, or didactic, way. Evidence of this might include paying close, exaggerated attention to phonetically difficult articulations, for example the pronunciation of [ɾ] (which is challenging in many languages, Bernhardt and Stemberger, 2018). They may also make their ADS cluster patterns more categorical in CDS to show the children how a particular cluster is typically produced and reduce ambiguity through making segment boundaries clearer.

Thus, there are various kinds of adjustments that one might expect in CDS, motivated potentially by different functions. These might affect incidence within and across different phonetic categories, and duration, differently, resulting in quite a complex picture. This is further complicated by the fact that mapping underlying motivations to observable behaviours may be ambiguous (i.e., a given behaviour, such as longer vocalic intrusions, may plausibly map onto more than one motivation) and the fact that underlying motivations are, of course, only speculative. Nevertheless, some plausible relationships can be posited. In Table 1, we set out a schematic overview of these for vocalic intrusions in Norwegian CDS, and suggest the potential phonetic evidence for these, as compared with ADS.

**TABLE 1:** Plausible underlying motivations for vocalic intrusions in CDS (compared to ADS) and their potential phonetic realisation.

Intention	Function	Potential evidence	Explanatory notes
Attracting attention	Bonding/Didactic (communicative)	Higher incidence, longer intrusions	Increase in acoustic salience
Expressing affect	Bonding	Higher incidence, longer intrusions	Increase in acoustic salience
Emphasising	Didactic (linguistic)	Higher incidence, longer intrusions	Selective increase in acoustic salience to facilitate word learning or comprehension of phrase structure
Instructing: mastering clusters	Didactic (phonetic)	Higher and/or more systematic incidence, longer intrusions	Exaggerating properties of ADS. Facilitates articulation of particular clusters (e.g., with rhotics)
Instructing: sounding ‘Norwegian’	Didactic (linguistic- phonetic)	Same incidence, longer intrusions	Patterning with, or exaggerating, ADS to help acquire Norwegian-appropriate cluster transitions (prosodic-phonetic biases)
Mirroring child speech patterns	Bonding, facilitating comprehension	Higher incidence, longer intrusions, more phonetic contexts	Patterning more closely with CS, changes as children develop

A strong prediction, across almost all kinds of possible motivating factors, is that vocalic intrusions will be of longer duration in CDS than in ADS. Both generalised properties of CDS, with universal communicative and bonding goals, and more didactically-orientated strategies are compatible with longer vocalic intrusions.

A slightly less strong prediction can be made about the incidence of vocalic intrusion in CDS when compared with ADS. Generalised CDS properties of slower tempo and exaggerated prosody would conspire to increase incidence, and this would be compatible with the general goals of attracting attention for increased affect and the conveyance of emotion, and for marking emphasis more transparently. It would also be compatible with the mirroring of child speech (for affect enhancement and facilitating comprehension), and for the phonetic didactic goal of instructing how to produce difficult clusters.

Finally, with respect to the distribution of vocalic intrusion in CDS, most potential motivating factors would predict little or no deviation from the distribution observed in ADS. There are two exceptions, however. Firstly, the goal of facilitating phonetic mastery of certain clusters, as well as highlighting the presence of the short [ɾ] as C2 in a cluster, that is, being didactic, could result in a higher incidence of intrusions in those specific phonetic contexts. Secondly, mirroring child speech patterns could result in a distribution pattern that deviates from ADS: Possible intrusions in CDS in phonetic contexts that are characteristic of child speech, but not of ADS, would be examples of adults mirroring the children in CDS, suggesting a bonding function, rather than a didactic one.

A further consideration is that the relationship between caregiver and child is dynamic, as are the communicative needs and abilities of the child. Thus tracing potential changes in CDS over a range of child ages can be enlightening. Knowing that prosodic characteristics of IDS and CDS appear to change over the age span of the infant/child being addressed, we expect vocalic intrusions to be less prominent in CDS directed toward older children than to younger ones.

With these considerations in mind, we analyse data from mothers speaking to 2;6-, 4- and 6-year-olds to investigate the following hypotheses:

(1) Vocalic intrusions have a higher incidence in CDS, at least when addressed to 2;6-year-olds, than in ADS.
(2) Vocalic intrusions have the same distribution with respect to phonetic contexts in CDS as in ADS.
(3) Vocalic intrusions are of longer duration in CDS, at least when addressed to 2;6-year-olds, than in ADS, but shorter than in child speech.
(4) Vocalic intrusions in CDS become less prevalent and of shorter duration as the children being addressed grow older.

## Materials and Methods

### Data

Our data consist of speech recordings from nine mothers reading a story to their child (CDS), compared to four mothers reading sentences to a research assistant (ADS), and nine children playing a naming game with their mothers, with words from the story they had just heard (CS). The ADS and CS data have already been reported on by Garmann et al. (2021), and serve as a basis for comparison here. The CDS, analysed specifically for this paper, was elicited from three Norwegian mothers of 2;6-year-olds, three mothers of 4-year-olds and three mothers of 6-year-olds. All nine mothers as well as their children were native speakers of Urban East Norwegian (Kristoffersen, 2000) and lived in and around Oslo. There is an overlap between three of the subjects providing ADS and CDS data, and between seven of the mothers and their children.

Audio was recorded using a Zoom Handy Recorder H2 with built-in microphones. The mothers and their children took part in a larger study comparing phonetic microvariation in the production of consonants in both child and adult speech in English and Norwegian, reported on by Payne et al. (2015, 2017) and Garmann et al. (2021). The studies were reviewed and approved by NSD – Norwegian Centre for Research Data under the reference number 36466. All participants or their legal guardians/next of kin provided written informed consent based on written and oral information.

To investigate cluster production, the materials were constructed around a set of target words with a variety of word initial consonant clusters. For ADS, the target words were included in a list of sentences, which the mothers were asked to read aloud to a researcher. To elicit CDS in a comparable setting, the target words were also included in a story of text and pictures made as a PowerPoint presentation. The CDS data consist of the speech of the mothers reading the story to their children. On the basis of this story, a naming game was constructed with pictures of the target words. The CS data consist of the children’s responses when playing the naming game with their mothers. For comparability, the CDS data for analysis were selected based on the CS cluster types analysed in Garmann et al. (2021). Table 2 shows the number of analysed instances across phonetic contexts and data sets.

**TABLE 2:** A breakdown of cluster types analysed in this paper, with incidences for each category of speech data.

Phonetic context		Clusters	CS data	CDS data	ADS data
Stop + liquid	Stop + /r/	/pr, br, tr, dr, kr, gr/	40	43	56
	Stop + /l/	/pl, bl, kl, gl/	24	19	32
Fricative (non-s) + liquid		/fl, fr/	25	21	16
S-clusters		/sp, st, sk, sl, sn, sm, sv/	67	60	72
Total			156	143	176

### Phonetic Analysis

Following Garmann et al. (2021), the productions were categorised into four groups, based on the occurrence of a (possible) vowel intrusion visible in spectrogram and waveform, as analysed by using the programme Praat (Boersma and Weenink, 2016). These four categories were defined in Garmann et al.(2021, p. 10) as:

(a) Clear vocalic intrusion: “a clearly definable period of high amplitude, voicing, formant structure, and of an easily measurable duration.”
(b) Relatively clear vocalic intrusion: “evidence of a post-consonantal period with lower amplitude than for (a), which may be either not fully voiced or with weak formant structure, and shorter in duration (or more difficult to measure) than for (a).”
(c) Possible ‘masked’ vocalic intrusion: “segment boundaries are hard to ascertain, e.g., because of a period of post-release aspiration and/or devoicing in an approximant may overlay a vocalic interval.”
(d) Definitely no vocalic intrusion: “no intervening acoustic material or discontinuity between C1 and C2.”

To assess the validity of this categorisation, 21% of the CDS words were blind-coded by a rater not involved in the original scoring. The agreement between raters concerning the existence of a vocalic intrusion (a + b vs. c + d) was 83%. For the ADS and CS, Garmann et al. (2021) reported a corresponding agreement of 75%. The duration of the vocalic intrusions in category (a) and (b) were measured in Praat, again following the methodology of Garmann et al. (2021).

To check for speech rate as a possibly confounding factor, we made a comparison between CDS and ADS: Garmann et al. (2021) measured the number of syllables per second in four identical ADS sentences as they were produced by three of the mothers of 2;6-year-olds. We now measured the number of syllables per second in four identical CDS sentences produced by the same three mothers. According to these measurements, the speech rate was significantly slower in CDS than in ADS (median 261 vs. 205 ms per syllable, *W* = 37.5, *p* = 0.0496), but there was no connection between these three mothers’ speech rate and the incidence or duration of vowel intrusions in their consonant clusters, as the mother with the slowest speech had just as many intrusions as the mother with the fastest speech, and only marginally longer intrusions (median 34 ms) than the two others (with a median of 31 and 32 ms, respectively).

### Statistical Analysis

Hypotheses regarding the incidence of vocalic intrusions [i.e., categories (a) and (b)] were tested with chi-squared (χ^2^) tests, or Fisher’s exact test for expected values at 4 or below. The hypotheses concerning the duration of vocalic intrusion were investigated using Wilcoxon rank sum tests, preferred over *t*-tests due to deviations from a normal distribution. These non-parametric statistical tests were deemed suitable for the data set consisting of 517 observations in total, with 192 observations from CDS compared to 159 from ADS and 156 from CS.

The study focuses on differences between samples rather than individual variation, based on Vihman et al. (1994), who found little individual differences at the segmental level within groups of mothers speaking the same language. In our study as mentioned above, all mothers spoke the same dialect, and the speech samples were scripted. Each mother’s speech showed considerable variation in the duration of vocalic intrusions, but there was no significant variation in neither incidence nor duration between the mothers: The incidence of vocalic intrusions did not differ significantly between the mothers with the most (MNR with 7 out of 10 measured clusters) and the fewest (HI with 6 out of 13) intrusions (*p* = 0.06), and there was no significant difference between the mothers with the longest (336 ms) and shortest (129 ms) median duration of intrusion (*W* = 25, *p* = 0.2).

The statistical analyses were carried out in R 4.0.2 (R Core Team., 2020) using RStudio 1.3 (RStudio Team., 2020). We used the stats package (R Core Team., 2020) to run tests and the package ggplot2 (Wickham, 2016) for visual inspection and preparation of figures.

## Results

### Categorisation

**FIGURE 1:**
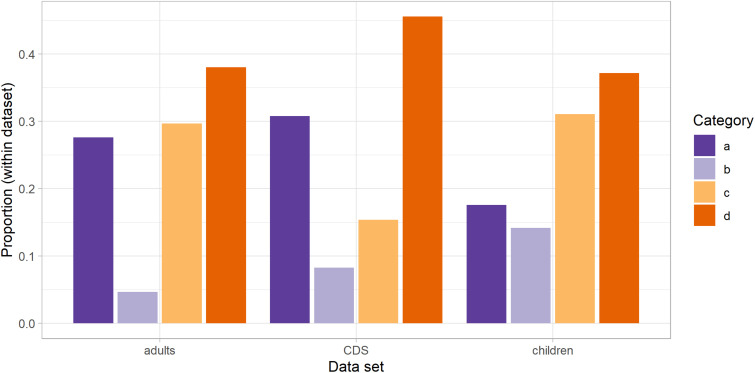
Distributions of the four categories of intrusion across the three datasets.

The proportions of the four categories of intrusion in the three datasets are shown in Figure 1. It is of note that category (c) constituted only 15% of the cluster productions in CDS, a significantly smaller proportion than Garmann et al. (2021) reported for ADS [30%, χ^2^(1) = 10.36, *p* = 0.001] and CS [29%, χ^2^(1) = 8.56, *p* = 0.003]. This category consists of the cases where segment boundaries could not be determined from the acoustic signal, due to the ambiguous alignment of certain articulatory parameters. For example, in a voiceless stop cluster + lateral, a medial period of voiceless aperiodicity could be attributed to post-aspiration of the voiceless stop, which could coincide with a (devoiced) vocalic intrusion, and/or a period of devoicing in the lateral.

The fact that this kind of ambiguous articulation was significantly reduced in CDS would suggest that adults avoided it, opting instead either for a more clear-cut open transition (and a definite, voiced period of vocalic intrusion), hence adding to categories (a) or (b), or potentially the reverse, suppressing post-release aspiration and therefore devoicing in a following lateral. As can be observed in Figure 1, the data suggest a greater inclination toward the latter: While all other categories [namely (a), (b), and (d)] have more incidences in CDS than in ADS, the difference is more notable for (d), although not significant in either. The decision was made to exclude category (c) from any further calculations, thus making the estimate of intrusion a conservative one (Garmann et al., 2021). However, this observed difference in how mothers categorise their articulations suggests a desire to avoid segmentally ‘ambiguous’ sequences (even if they are completely natural in ADS).

### Incidence

With regard to the incidence of vocalic intrusion, there was no significant difference between CDS (46.1%) and ADS [46.9%, χ^2^(1) = 0, *p* = 1], when viewed overall. Following the assumption that traits of CDS are more likely to be present in speech addressed to younger children, we isolated the CDS directed toward the 2;6-year-olds and compared this to ADS. Still, there was no significant difference in incidence [χ^2^(1) = 0.724, *p* = 0.395]. This means that, viewed across phonetic contexts, and compared with ADS, CDS does not have more vocalic intrusion than ADS.

Next, we investigated potential differences in the incidence of vocalic intrusion in the CDS by child age. The adults addressing 2;6-year-olds had vocalic intrusion in 53% of their consonant clusters, while the adults speaking to the older children produced 40% of their consonant clusters with a vocalic intrusion. According to a chi-squared test, this difference was significant [χ^2^(1) = 5.17, *p* = 0.023]. There were no significant differences in the number of intrusions between adults addressing 4-year-olds (42%) compared to adults addressing 6-year-olds (38%). Thus, while the differences are not sufficient to make a significant difference when compared with ADS, there is evidence that mothers are behaving differently toward 2;6 year-olds, when compared with 4 and 6 year-olds.

Comparing children’s speech and CDS within each age group (see Table 3), we found a significant difference in incidence between mothers and children at 2;6 [χ^2^(1) = 5.14, *p* = 0.023], but not at 4 [χ^2^(1) = 1.35, *p* = 0.245] or 6 [χ^2^(1) = 0.20, *p* = 0.6543]. The figures indicate a correspondence between CDS and CS, but with a delay: The proportions of vocalic intrusions in children’s speech at 4 and 6 resemble the proportions in CDS at 2;6 and 4, respectively (For a discussion of the development of vocalic intrusion in CS, cf. Garmann et al., 2021).

**TABLE 3:** Number and proportion of vocalic intrusions in produced clusters in CDS and CS by age of the child.

Child age	CDS	CS
2;6	23/36 (64%)	9/28 (32%)
4	24/57 (42%)	23/41 (56%)
6	19/50 (38%)	15/33 (45%)

### Distribution by Cluster Type

As shown in Table 4, the vocalic intrusions generally occurred in the same phonetic contexts in CDS and in ADS, but CDS also had a few intrusions in non-liquid contexts. There were no significant differences between the two datasets in the incidence of intrusions when the second consonant was a non-liquid (*p* = 1 according to Fisher’s exact test), or a lateral [χ^2^(1) = 0.04, *p* = 0.844]. However, intrusions were significantly more common in CDS than in ADS when the second consonant was a tap or a flap [χ^2^(1) = 5.67, *p* = 0.017]. There was only one occurrence in CDS of a consonant cluster with a tap or flap as C2 produced without a vocalic intrusion, namely a production of *krakk* ‘stool’ addressed to a 6-year-old. In other words, in CDS there is no apparent increased incidence of vocalic intrusions when viewed across phonetic contexts, but there does appear to be a greater incidence within a specific context, namely pre-rhotic. It should be noted that vocalic intrusion is almost omnipresent in this context. Thus, adults are differentiating their speech toward children in terms of extent of vocalic intrusion for a given phonetic context, making something that occurs gradiently in ADS, categorically occurring in CDS. In other words, it is a more systematic feature of CDS than of ADS.

**TABLE 4:** The number of instances with a vowel intrusion and the number of clusters measured in the three data sets, by C2 category.

	C2 non-liquid	C2 lateral	C2 tap/flap	Total
CS: intrusions	10/60 (17%)	9/11 (82%)	28/31 (90%)	36/102 (35%)
CDS: intrusions	7/60 (12%)	7/30 (23%)	52/53 (98%)	66/143 (46%)
ADS: intrusions	0/43 (0%)	6/20 (30%)	54/65 (83%)	60/128 (47%)

While we found no vocalic intrusions at all in non-liquid C2 clusters in ADS, in effect /sC/ clusters, there were 7 occurrences in this phonetic context in the CDS data, in the words *smokk* ‘pacifier,’ *snørr* ‘snot,’ *svane* ‘swan,’ and *sverd* ‘sword.’ These instances were evenly distributed across age groups. The difference between CDS and ADS is, as noted above, not statistically significant, but the mere occurrence is interesting, particularly because we also saw intrusions in this context in children’s speech, as shown in Table 4. This indicates that mothers may be mirroring their children when it comes to phonetic contexts. There was no significant difference between incidence in CDS and children’s speech when the C2 was a non-liquid [χ^2^(1) = 0.27, *p* = 0.600] or a tap/flap [χ^2^(1) = 1.18, *p* = 0.277], but vocalic intrusions were significantly less common in CDS than in CS when the second consonant was a lateral [χ^2^(1) = 9.24, *p* = 0.002].

### Duration

**FIGURE 2:**
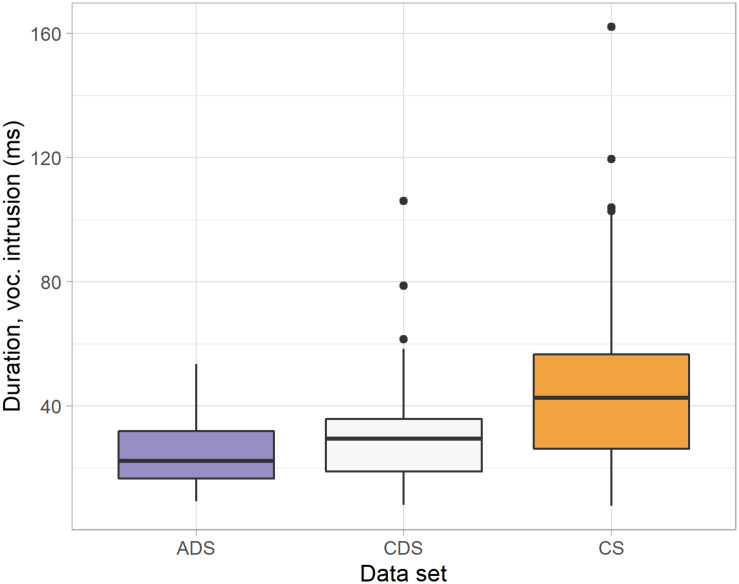
The duration of vocalic intrusions in consonant clusters produced in ADS, CDS, and CS.

**FIGURE 3:**
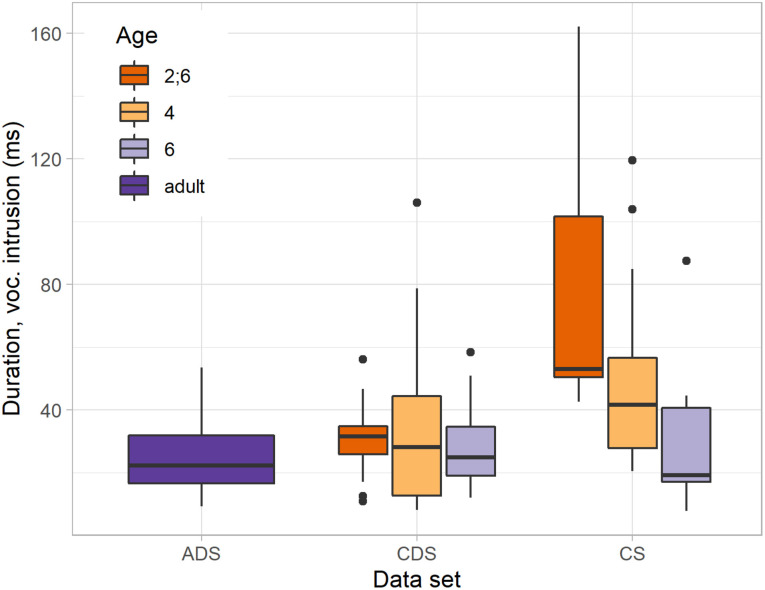
Duration of vocalic intrusion in ADS, in CDS to different age groups (2;6, 4, and 6) and in CS in different age groups (2;6, 4, and 6).

Turning to the duration of the vocalic intrusions, there was no significant difference between CDS (median = 29 ms), when viewed overall, and ADS (median = 22 ms, *W* = 1390, *p* = 0.078), as shown in Figure 2. However, when we looked specifically at the speech directed toward the youngest children, the difference between CDS and ADS *was* significant: The intrusions in CDS were significantly longer when adults addressed 2;6-year-olds (median = 32 ms) than when adults addressed adults (*W* = 804, *p* = 0.018), see Figure 3. This difference was not found when comparing ADS with CDS addressed to 4-year-olds (median = 28 ms, *W* = 658, *p* = 0.708) or 6-year-olds (median = 25 ms, *W* = 580, *p* = 0.267). Thus, in terms of *duration of intrusion*, adults are differentiating their CDS as a function of the age of the child being addressed. In other words, vocalic intrusion is arguably a more *salient* feature of CDS addressed to very young children.

Looking at the duration of vocalic intrusions between CDS overall (median = 29 ms) and CS overall (median = 43 ms), the former was significantly shorter than the latter (*W* = 811, *p* < 0.001). As can be seen from Figure 2, there may be a development corresponding to what occurs in children’s speech, where Garmann et al. (2021) reported a significant reduction in duration with age. However, there is a lot of variation masking possible changes in CDS over time: Investigating the duration of vocalic intrusions in the CDS data divided by the children’s age, we found no significant differences between the CDS addressed to 2;6-, 4-, and 6-year-olds. Children and mothers appear to approach each other over time: There was a significant difference in duration of the vocalic insertions between CDS to 2;6-year-olds (median = 32 ms) and the 2;6-year-olds themselves (median = 53 ms, *W* = 201, *p* < 0.001), and a smaller, but still significant difference between mothers (median = 28 ms) and children (median = 41 ms) at 4 years (*W* = 358, *p* = 0.015), but no difference between mothers (median = 25 ms) and children (median = 19 ms) at age 6 (*W* = 92, *p* = 0.611).

## Discussion

In this paper, we set out to study vocalic intrusions in the production of clusters in Norwegian CDS. This prosodic-phonetic bias has been observed in Norwegian ADS as well as in CS. We investigated whether vocalic intrusions are present also in CDS, and if so, whether they occur with the same incidence and patterns of distribution. We analysed CDS from mothers addressed to children aged 2;6, 4, and 6, and compared these data to ADS and CS data collected and analysed by Garmann et al. (2021). Furthermore, we divided the CDS data by age to look for changes as children grow older. We tested four hypotheses, namely that vocalic intrusions (1) have a higher incidence in CDS than in ADS; (2) have the same distribution with respect to phonetic contexts in CDS as in ADS; (3) are of longer duration in CDS than in ADS but shorter than in children’s speech, and (4) their incidence and duration in CDS diminish as children grow older.

Regarding our first hypothesis, we found that when viewed overall, there is the same prevalence of vocalic intrusions in CDS and ADS. This suggests that increasing the incidence of vocalic intrusion is not used as a generalised strategy either to increase affect or bonding or for didactic purposes. However, when isolating the speech addressed to the youngest children, we did find intrusions to be significantly more common than in ADS. We interpret this as indicating that bonding strategies concerned with attracting attention, conveying affect and mirroring the child’s speech behaviour, together with didactic goals of instructing how to convey emphasis and to produce difficult clusters, are more important in speech addressed to the younger children.

Moreover, we found a significantly smaller proportion of clusters analysed as category (c) clusters (i.e., clusters where the segment boundaries are hard to identify) in CDS when compared to ADS. This indicates that even if the incidence of intrusions generally is the same in CDS and ADS, mothers tend to produce their clusters more clearly either with or without intrusions when speaking to children. In other words, there is evidence that some adjustments may be made to clarify an ambiguous segmental boundary within a cluster, which may play some phonological didactic function in terms of reinforcing the segmental composition of a sequence.

Concerning our second hypothesis, the vocalic intrusions are generally found in the same phonetic contexts in CDS and ADS, with two exceptions: Firstly, albeit only marginally, CDS contained instances of vocalic intrusion in non-liquid /sC/ clusters, which to some extent mirrors the greater cross-context generalisation of this feature in child speech. In addition to possible mirroring of child speech behaviour, this shows an *expansion* in the range of incidence, even if the overall level of incidence is the same. Perhaps more important, however, is the finding that vocalic intrusions were significantly more common (and indeed almost entirely categorically present) in CDS than ADS in clusters that could be considered to be particularly challenging for the child, that is when the second consonant was a tap or a flap (e.g., in *brannmann* ‘fireman,’ [ˈbɾɑn.mɑn], and *glass* ‘glass,’ [ˈɡɽɑs]). This suggests that while vocalic intrusion may not be more prevalent overall in CDS, it does appear to be *systematically* applied in those phonetic contexts in which it is frequently – though not categorically – applied in ADS. In view of the facts that the Norwegian tap [ɾ] is short and can be difficult to perceive without a preceding intrusion (Bradley, 2007) and being one of the latest speech sounds to be acquired by children speaking Urban East Norwegian (Fintoft et al., 1983), this would appear to be motivated by a phonetic didactic intent.

When it comes to the third hypothesis, a longer duration in vocalic intrusion was strongly predicted, being compatible with a number of possible motivations. Longer durations are associated with greater acoustic salience, which are compatible with general strategies for attracting more attention and conveying affect. The strongest version of this hypothesis was not confirmed, as there were no significant differences in the duration of intrusions between CDS in general and ADS, but the intrusions had longer durations in CDS addressed to 2;6-year-olds than in ADS. Our interpretation is that this particular modification is primarily underpinned by bonding strategies, since Wang et al. (2018) have suggested that the purpose of IDS directed toward younger infants is more geared toward increasing closeness. There may also be some didactic intent in drawing attention to specific words by emphasising them. If there were some form of didactic intent related to cluster production specifically, we might expect it to persist in CDS directed toward older children, but this appears not to be the case. We also note that while vocalic intrusions are longer in CS than in either CDS or ADS, they are particularly long in the speech of 2;6-year-olds, hence providing possible evidence for a (albeit somewhat subtle) mirroring effect in the CDS addressed to that age group. We interpret a mirroring behaviour as another bonding strategy, since it seeks to close the distance between the behaviour of the child and that of the speaker. In mirroring articulation strategies of the child, it also arguably facilitates comprehension. While longer intrusions are compatible with various didactic intents (e.g., showing higher linguistic structure such as emphasis, or showing the linguistic-phonetic detail of how to ‘sound Norwegian,’ or simply to aid phonetic mastery of a universally difficult articulation), we would expect these to be employed also, or even especially, with older children, which is not the case. We thus conclude that longer vocalic intrusions are principally a bonding strategy with possibly a didactic function of word learning, employed selectively toward the youngest children.

Thus far, it would appear that mothers do indeed modify their productions of consonant clusters, and in a number of quite specific ways. Firstly, they reduce the proportion of clusters with ambiguous segmental boundaries [category (c) clusters]. Secondly, they systematise a phonetically natural tendency within a specific category of clusters (those with a rhotic C2), and marginally extend this tendency to other phonetic contexts (i.e., with a non-liquid). Thirdly, they phonetically exaggerate the intrusion, through lengthening, to children under 4 years only. From the literature, we know that we adapt our speech to our interlocutors in myriad ways, and that different properties of CDS may fade at different stages, thus revealing complex strategic modification of speech in parent–child interactions. Recall that Payne et al. (2015) found that mothers modified their rhythm in CDS to children aged 2–6, with no change over time, while Warren-Leubecker and Bohannon (1984) found fathers to use a higher pitch and wider pitch range when speaking to 2-year-olds, but not to 5-year-olds. The mothers in the same study adapted in both pitch and pitch range to both 2- and 5-year-olds.

This marginal extension and exaggeration of intrusions in CDS appears to echo reported patterns in CS in which we found that intrusions were also generalised beyond the ADS phonetic contexts (Garmann et al., 2021). A critical question is whether children are simply replicating this pattern in the CDS input, that is, their behaviour is driven by phonetic evidence in the input, or if they are in fact going beyond this and imposing their own structural constraints on their output. If they extend and/or exaggerate significantly more than in CDS, that would indicate a degree of abstract mediation, along the lines of Vihman and Velleman (2000). Further research, comparing CS and CDS more directly and for a wider range of discourse contexts is needed to establish this.

As for the fourth hypothesis concerning a decline of both incidence and duration of intrusions as children grow older, we found a higher incidence in speech addressed to 2;6-year-olds than to the older children, but no significant difference in duration of the intrusions. Comparing mothers and children, we found significantly more vocalic intrusions among the children at age 2;6, but no significant difference at age 4 or 6. Correspondingly, the intrusions were significantly shorter in CDS than in CS at age 2;6 and 4 (although with a smaller difference), while the difference had disappeared completely at age 6. Hence, concerning vocalic intrusions in Norwegian, it appears that exaggerations in incidence and duration are a property of CDS addressed to young children only. Why does this property of CDS disappear so early?

The finding is in line with those of Warren-Leubecker and Bohannon (1984) for pitch qualities in fathers’ speech. While the function of speech addressed to infants (IDS) may be to draw the child’s attention, speech addressed to children above 2 years (CDS) may have more of a didactic function, where more distinct speech may support children in the further segmentation of fluent speech (Fernald and Mazzie, 1991). It is, however, difficult to say which function the CDS to the 2;6-year-olds serve, which is different from 4- to 6-year-olds. It could be that some of the explanation can be found in the setting studied in this paper, namely reading a story. It is likely that 2;6-year-olds have more difficulties in focussing on the reading task than the older children, which may suggest that the mothers tried to keep their children’s attention. On the other hand, it could also be that the mothers subconsciously aim to help the children in recognising clusters or perceiving their constituents, and that the CDS in this situation therefore had a more didactic function. The mothers were telling a picture-based story presenting words that they perhaps were not certain that their children knew, preparing them for a later naming task (see Garmann et al., 2021). In support of the didactic function of CDS, the mothers of 2;6-year-olds addressed their children with a frequency and duration of vocalic intrusions similar to the productions of 4-year-olds, and correspondingly, the 4-year-olds received input similar to the productions of 6-year-olds. Hence, the mothers may be guiding their children to more ADS-like speech, being one step ahead of their children.

As we have noted in the introduction, there are few studies reporting on CDS to children older than 2, and even fewer reporting on the path that caregivers take, modifying the details of their CDS register to move away from child-directed adaptations. Thus, more is known about the transition from IDS to CDS than about modifications within later stages of CDS, or indeed about the transition from CDS to ADS. Our study has made a contribution by establishing that the incidence of vocalic intrusions is higher and the duration is longer in CDS addressed to 2;6-year-olds than to older children as well as adults, and that compared to ADS, Norwegian CDS is at the same time both more systematic (near-obligatory vocalic intrusions before taps and flaps) and closer to child speech (producing vocalic intrusions also in non-liquid /sC/ clusters). Finally, mothers appear to guide their children by mirroring them while at the same time staying one step ahead.

In the Section “Introduction,” we outlined a range of different intentions that mothers may have in adapting their speech to children, and two possible underlying functions: bonding and didactic. Most – though not all – of the behaviours underpinned by these intentions pull in the same direction, namely to a higher incidence and longer duration of vocalic intrusions in CDS, contributing to an increase in acoustic salience. Hence, these two properties may fill multiple intentions and underlying functions at the same time. As they are only present in speech addressed to the 2;6-year-olds, it is possible that the specific intentions of attracting attention and instructing sounding Norwegian lose their importance some time before age 4.

There is evidence that other behaviours persist beyond this age. Two of the intentions discussed in the introduction appear to be at play throughout the age range in this study, namely instructing the mastery of Norwegian clusters, with a didactic function, and mirroring children, with a bonding function. These two pull in different directions. From a phonetic didactic point of view, we would expect CDS to be more systematic. However, for the purpose of bonding, mothers may subconsciously try to mirror their children’s speech patterns, producing intrusions also where they do not occur in ADS. We see both these functions at play in our data. First, the CDS data we have reported here very clearly show that mothers are much more unambiguously employing vocalic intrusion in *specific* phonetic contexts, and overwhelmingly in the pre-tap/flap context. By increasing the systematicity of vocalic intrusion for this category of cluster, they are arguably driving this home even more categorically, especially if we consider that potentially ambiguously segmented clusters [category (c)] are resolved more definitively as category (d) productions. However, we also see seven intrusions in CDS in one phonetic context where it occurs in CS but not ADS, namely in non-liquid clusters. The mere existence, even if they are few, suggests mirroring, and by that a bonding function possibly accompanied by a desire to facilitate comprehension. Note that the fact that the systematicity does not change over the time period that we have studied would suggest that it has a different function from the increased phonetic salience of the longer duration of vocalic intrusion in speech addressed to 2;6-year-olds.

## Conclusion

We advocate a wider approach to the investigation of dynamic variations according to individual and interactional factors, to extend to pervasive characteristics of intersegmental timing and coordination. Further investigation is needed to consider how this approach could be integrated into existing dynamic interactive models of phonological acquisition (cf. Vihman and Velleman, 2000; Davis and Bedore, 2013). Vocalic intrusions in consonant clusters are a property, or prosodic-phonetic bias, of Norwegian (Garmann et al., 2021), as a language with an open transition between the consonants in a cluster (Endresen, 1991). It would be interesting to see whether this phenomenon is treated similarly by parents speaking for example Bulgarian or Portuguese to their children, since children speaking these languages produce vocalic intrusions in clusters (Ignatova et al., 2018; Ramalho and Freitas, 2018). This paper has shown how this particular prosodic-phonetic bias is subject to quite detailed and stratified modification in CDS, arguably with multiple functions. It thus highlights the potential importance of such biases to adaptive speech variation used in a variety of discourse contexts, and for different purposes. For example, we might conceive of increased vocalic intrusion as a strategy of hyperarticulation. To increase our understanding of the function that this property has in increasing comprehension, it would be interesting to investigate consonant clusters in speech directed to other groups for whom register adaptations have been observed, for example as L2 learners, elderly persons or individuals with receptive language difficulties, or for speech in poor listening conditions. More broadly, we advocate for an approach that incorporates three fundamental aspects of CDS. Firstly, CDS is *dynamic*, and we thus need to differentiate by child age, and to trace a longer trajectory. Secondly, CDS is *complex*, and thus we need to tease apart different aspects of speech (e.g., phonetic vs. phonological, segmental vs. longer domain aspects of connected speech). And finally, the relationship between CDS and CS is *interactive*, and to properly understand this requires close analysis of CDS in relation to child speech. This encompasses both the possible interaction of bonding and didactic functions in the dyadic relationship, and the role that phonetic-prosodic biases in the implementation of phonology in CDS, play in the construction of the child’s phonology.

## Study Limitations

We have looked at CDS, and compared it with ADS and CS, within a specific and limited interaction context. Although we tried to make the elicitation situation as similar as possible between data sets, the different reading contexts for ADS and CDS are not identical. The sample in the study is relatively small, so the results should be interpreted with caution. A larger data set with more participants covering other situations might yield different patterns of vocalic intrusions in CDS and would also lead to better possibilities for generalisation. Ideally, we would also have included CDS and ADS data from all the same mothers, as well as CS data from their children to reinvestigate the conclusion in Vihman et al. (1994) that there is little individual variation between mothers as to segmental properties of CDS. Furthermore, we found variation in both speech rate as well as incidence and duration of vocalic intrusions between the mothers while speaking to their children, but no connections between speech rate and intrusions. However, as the data set was limited in size, we cannot rule out the possibilities of such connections. Moreover, adding another data point between age 2 and 3 could shed light on when a higher incidence and a longer duration of vowel intrusions fade in CDS; it can furthermore be interesting to study CDS to children who are older than 6 years of age.

We found that mothers adapted to their child’s language level with age, but having more detailed knowledge about the children’s language skills could have informed us on how parents adjust the proportion of and phonetic contexts in which they produce vocalic intrusions in consonant clusters. Whereas our data were cross-sectional, a longitudinal study of children in the age range 2–6 or older could also tell us more about the individual ways in which we adapt to our children, and whether there is a correspondence between the systematicity of vocalic intrusions before taps in individual parents’ speech and their children’s mastery of the tap. This would align our work with an increasing body of research which shows that young children are sensitive to variable acoustic information in the input that is non-phonemic, and that this sensitivity is reflected in their production (e.g., coda stop release in Sim and Post, under review, and vocalic intrusion in clusters, here), highlighting the importance of the precise quality of the input, in addition to the quantity of patterns of realisation.

As Warren-Leubecker and Bohannon (1984) found differences between mothers and fathers with respect to high pitch and pitch variation, it could also be interesting to investigate all potential characteristics of CDS in both mothers and fathers. Nevertheless, the lion’s share of the research on CDS since has focussed on mothers’, or female caregivers’, speech. There is a clear need for also including fathers/male caregivers in future research and investigating the variable of gender of the child being addressed. Moreover, we do not know whether there are differences between parents depending on social factors like maternal education. A further variable of potential interest is sibling order and the degree to which infants/young children are also communicating with each other, and whether this ‘child input’ further affects the trajectory of children’s articulatory fine-tuning.

## Data Availability Statement

The datasets generated for this study are not readily available because of ethical and legal restrictions related to sound files. Requests to access the datasets should be directed to NG.

## Ethics Statement

The studies involving human participants were reviewed and approved by NSD – Norwegian Centre for Research Data. Written informed consent to participate in this study was provided by the participants’ legal guardian/next of kin.

## Author Contributions

EP and BP initiated the ACT-project within which this manuscript is situated. NG, PH, and HS contributed to the conception and design of this particular study and organised the data collection and data analyses in Praat. NG obtained financing for the Norwegian analyses. ET and EH analysed the data in Praat and participated in discussions of research questions, analyses and categorisation of data. PH performed the statistical analyses. With the help of PH, NG, HS, ET, and EH prepared and held a conference presentation on the study. Together with NG, PH, HS, EP, and BP prepared the data collection and EP trained research assistants, as well as EH and ET, in analysing the data. NG, PH, and HS wrote the first and second drafts of the manuscript and finalised it. EP and BP contributed with considerable portions of text to the first and second drafts. ET and NG edited the final text. All authors contributed to manuscript revision, read, and approved the submitted version.

## Conflict of Interest

The authors declare that the research was conducted in the absence of any commercial or financial relationships that could be construed as a potential conflict of interest.
