# Supplementary material for: Vocalic Intrusions in Consonant Clusters in Child-Directed vs. Adult-Directed Speech
Source: Front Psychol. 2021 Jul 19;12:688002. doi: 10.3389/fpsyg.2021.688002 (PMC8326906; doi:10.3389/fpsyg.2021.688002)
Supplement: Supplementary file 1 [file Data_Sheet_1.docx]

Supplementary Material

# Supplementary Tables

**Supplementary Table 1.** Number of words with consonant clusters according to cluster type in adult directed speech.

| ADS | |  |  |
| --- | --- | --- | --- |
| Context | | Clusters | Number of words |
| Stop + liquid | Stop + /r/ | /pr, br, tr, dr, kr, gr/ | 56 |
|  | Stop + /l/ | /pl, bl, kl, gl/ | 32 |
| Fricative (non–s) + liquid |  | /fl, fr/ | 16 |
| S-clusters |  | /sp, st, sk, sn, sm, sv, sl/ | 72 |
| Total |  |  | 176 |

**Supplementary Table 2.** Number of words with consonant clusters according to cluster type in child speech.

| CS | |  |  |
| --- | --- | --- | --- |
| Context | | Clusters | Number of words |
| Stop + liquid | Stop + /r/ | /br, tr, kr/ | 40 |
|  | Stop + /l/ | /pl, kl, gl/ | 24 |
| Fricative (non–s) + liquid |  | /fl, fr/ | 25 |
| S-clusters |  | /sp, st, sk, sn, sm, sv/ | 67 |
| Total |  |  | 156 |

**Supplementary Table 3.** Number of words with consonant clusters according to cluster type in child directed speech.

| CDS | |  |  |
| --- | --- | --- | --- |
| Context | | Clusters | Number of words |
| Stop + liquid | Stop + /r/ | /br, tr, kr/ | 43 |
|  | Stop + /l/ | /pl, kl, gl/ | 19 |
| Fricative (non–s) + liquid |  | /fl, fr/ | 21 |
| S-clusters |  | /sp, st, sk, sn, sm, sv/ | 60 |
| Total |  |  | 143 |
